# Role of Sediments in Insecticide Runoff from Urban Surfaces: Analysis and Modeling

**DOI:** 10.3390/ijerph15071464

**Published:** 2018-07-11

**Authors:** Angela Gorgoglione, Fabián A. Bombardelli, Bruno J. L. Pitton, Lorence R. Oki, Darren L. Haver, Thomas M. Young

**Affiliations:** 1 Department of Civil and Environmental Engineering, University of California, Davis, One Shields Avenue, Davis, CA 95616, USA; agorgoglione@ucdavis.edu (A.G.); fabombardelli@ucdavis.edu (F.A.B.); 2 Department of Plant Sciences, University of California, Davis, One Shields Avenue, Davis, CA 95616, USA; bjpitton@ucdavis.edu (B.J.L.P.); lroki@ucdavis.edu (L.R.O.); 3 Division of Agriculture and Natural Resources, South Coast Research & Extension Center, University of California, Irvine, 7601 Irvine Blvd., Irvine, CA 92618, USA; dlhaver@ucanr.edus

**Keywords:** build-up, pyrethroids, SWMM, total suspended solids, wash-off

## Abstract

Insecticides, such as pyrethroids, have frequently been detected in runoff from urban areas, and their offsite transport can cause aquatic toxicity in urban streams and estuaries. To better understand the wash-off process of pesticide residues in urban runoff, the association of pyrethroids with sediment in runoff from residential surfaces was investigated in two watersheds located in Northern California (Sacramento County). Rainfall, flow rate, and event mean concentrations/loads of sediments and pyrethroids, collected during seasonal monitoring campaigns from 2007 to 2014, were analyzed to identify relationships among stormwater quality and rainfall characteristics, primarily using Principal Component Analysis (PCA). Pyrethroid wash-off was strongly related to sediment wash-off whenever sediment loads exceeded 10 mg; this value was conveniently selected as a threshold between dissolved and particle-bound control of off-site pyrethroid transport. A new mechanistic model for predicting pyrethroid wash-off profiles from residential surfaces at basin-scale was implemented in the Storm Water Management Model (SWMM). The accuracy of the model predictions was estimated by evaluating the root mean square error (RMSE), Nash–Sutcliff efficiency (NSE), and Kling–Gupta efficiency (KGE) for each pyrethroid detected (RMSE_tot_ = 0.13; NSE_tot_ = 0.28; KGE_tot_ = 0.56). The importance of particle-bound transport revealed in this work confirms previous field investigations at a smaller scale, and it should be a key consideration when developing policies to mitigate pesticide runoff from urban areas.

## 1. Introduction

Insecticides commonly used for urban structural pest control and landscape maintenance, such as synthetic pyrethroids, have been identified as a leading cause of invertebrate toxicity in water and sediments of urban streams and estuaries around the United States, especially in California [1,2,3]. For this reason, many studies have classified pesticide use in urban regions as a contribution to non-point source pollution to surface water [4,5]. Offsite movement of insecticides applied around residential homes, facilitated by irrigation and rain-induced surface runoff over impervious urban surfaces, has been identified as a primary source of pollution in urban watersheds [6,7,8].

It is well-known that synthetic pyrethroids have a strong affinity for surfaces due to their high hydrophobicity, as reflected by large octanol-water partitioning coefficient (K_ow_) values; also adsorption to loose soil or sediment particles is the primary form by which their offsite movement takes place in soil-water or sediment-water continuums [9]. Therefore, knowing that impervious surfaces are not generally cleaned prior to pesticide applications, it is realistic to assume that there is a direct application of active ingredients to particulate matter on the surface [10].

In the past, researchers focused on the environmental fate of pesticides in different matrices such as soil and water. Only recently have studies been designed to better understand pesticide behaviors on urban surfaces and the transferability of pesticide residues from residential surfaces to runoff water [6,7,11,12]. Among these, only a few studies developed a mathematical model able to simulate pesticide wash-off in urban areas by considering dissolution as the primary mechanism by which pyrethroid insecticides are transported away from their point of application [13,14]. However, it is noteworthy that previous research regarding the occurrence, characterization, and prediction of insecticide wash-off from urban surfaces has almost exclusively been supported by small-scale experiments, such as those on concrete cubes or slabs, with pesticide spikes and simulated rainfall. As far as we know, this manuscript represents the first study that creates a new mechanistic model for predicting pesticide wash-off from residential surfaces as a function of sediment wash-off, and deals with the challenges of basin-scale modeling resulting from the uncertainty in the input parameters (e.g., mass of pesticides applied on the surface, cleaning of the surfaces, amount of pesticide available to runoff extraction, etc.).

The primary objectives of this study were to: (i) understand and analyze the role of sediments in the transport of pesticides from residential surfaces in real urban areas (basin-scale); and (ii) build and test a new physically-based model able to mathematically characterize and predict the transport of pesticides from residential surfaces on the basis of sediment wash-off, by exploiting the outcomes obtained from objective (i).

Two watersheds in Northern California were monitored during multiple storms between 2007 and 2014, providing records of stormwater runoff quality (bifenthrin, cyfluthrin, cypermethrin, and sediment Event Mean Concentration (EMC)), runoff quantity (flow rate) and rainfall. These data were analyzed with a multivariate statistical method to correlate pesticide accumulation and runoff with different rainfall characteristics and sediment build-up and wash-off processes [15,16]. The use of this type of analysis helps to overcome significant limitations that may be created by looking at each variable independently without having a holistic view and an in-depth understanding of the entire system. From the observed outcomes, a mathematical model was developed for predicting pyrethroid wash-off based on sediment wash-off. By assessing model performance and interpreting model parameters, this study provides, in our opinion, one of the first comprehensive approaches for the numerical prediction of pesticide wash-off from residential surfaces based on sediment wash-off. This model incorporates a typical Californian land-cover of a low and very-low-density residential area; therefore, it is highly applicable to most of the other residential areas in the state. The results will contribute to the quantification of pesticide risks in urban environmental settings, and inform appropriate mitigation policies to control those risks.

## 2. Materials and Methods

### 2.1. Study Sites

**Figure 1:**
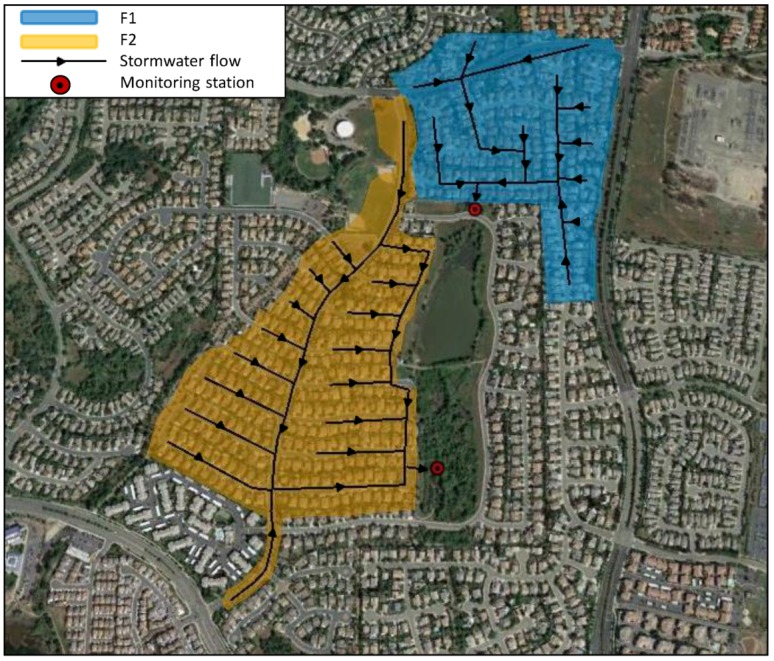
Study watersheds with the location of their monitoring stations and stormwater flow directions. (Adapted from Google Earth https://earth.google.com/web/).

The study area includes two urban watersheds located in Folsom, Sacramento County, CA, USA. The two basins, called Folsom 1 (F1) and Folsom 2 (F2) in the current work, are adjacent but physically separated by some terrain features and lack of connecting roadways. The F1 drainage area has a surface area of 21,634 m^2^ with 49.6% impervious surface, while F2 has a surface area of 30,202 m^2^, with 52.4% impervious surface. In both basins, the stormwater flows through a separate drainage network that ends with a 1.22 m cement culvert. In Figure 1, we show the two catchments, the locations of the monitoring stations and stormwater flow directions.

### 2.2. Data Collection

#### 2.2.1. Monitoring Campaign

The two catchments have been continuously monitored for selected stormwater quality parameters, flows, and rainfall quantities using automatic monitoring stations established at the outlets. In particular, a bubble level meter and an auto-sampler were installed to measure flow rate and to collect samples for total suspended solids (TSS) and insecticide concentration analyses [17]. The flow meter sensor directly provides the water depth in the runoff channel with an accuracy of +/−0.033 m and the average velocity of the flow stream by using an acoustic Doppler velocimeter; it calculates flow based on the current level and the definition of discharge (Wetted Area × Velocity = Flow). Water quality data collection was programmed by auto-samplers at regular intervals. To ensure the ground was sufficiently wet to generate runoff, 0.20 cm of rainfall was necessary to trigger sampling for the first storm event of the season. The pacing volumes used to program the auto-samplers were based on collecting 50 samples of 400 mL each, resulting in a total of 20 L. However, the autosamplers were actually programmed to collect 75 samples, in case the storm duration or intensity was greater than predicted.

The monitoring campaign comprised 11 storm events at F1 and 20 storm events at F2 from 2007 to 2014. Rainfall/runoff data relating to all the events, including total rainfall depth (mm), rainfall event duration (h), duration of antecedent dry weather (day), total runoff volume (m^3^), runoff peak flow rate (m^3^/s), and total depth measured at the outfall (m) are summarized in the Supplementary Materials (SM-1). Stormwater quality data were monitored for 17 events from 2007 to 2010 by measuring the EMC of total TSS and selected insecticides: bifenthrin, cyfluthrin, and cypermethrin. This selection was made on the basis of the aquatic toxicity, amounts of use, and detection frequency in urban and residential areas of California of the pyrethroids chosen [10,13]. The EMC of each parameter is defined as follows:

(1) $$\mathrm{EMC}=\frac{\sum_{i=1}^{n}C_{i}V_{i}}{V}$$

where *V* is the total runoff volume per event (L), *V_i_* is the runoff volume during time increment *i* (L), *C_i_* is the average pollutant concentration during time step *i* (mg/L), and *n* is the total number of samples collected during a single storm event; the event mean load (EML) is defined as the total mass of each constituent that ran off during a particular event; it is calculated as:

(2) $$\mathrm{EML}=\sum_{i}C_{i}V_{i}=\mathrm{EMC}\cdot V$$

TSS and pyrethroid EML values are listed in the Supplementary Materials (SM-2).

#### 2.2.2. Pesticide Use Report (PUR) Database

Pyrethroid insecticide application data for 2007 through 2010 was downloaded from the Pesticide Use Reporting (PUR) database maintained by the California Department of Pesticide Regulation (http://calpip.cdpr.ca.gov/main.cfm). To organize the pesticide-use data for model input, a criterion to filter and discard records in the PUR database was applied [18]. Data for Sacramento County were filtered to obtain only structural pest control and landscape maintenance entries for pyrethroid active ingredients. In the PUR database, these entries are usually dated as the first of the month. Therefore, by assuming that the amount of pesticide used every day is constant, a formula to estimate the average daily amount of insecticide applied on F1 and F2 was developed (Equation (3)):

(3) $$M_{app\_pest}=\frac{\left(\sum PC_{x}\;\right)\cdot \left(\frac{A_{F}}{A_{S}}\right)}{n_{x}}$$

where *PC_x_* is the mass of chemical applied daily in the month *x*; *n_x_* is the number of days of month *x*; *A_F_* is the area of the watershed (F1 or F2); and *A_S_* is the total developed area of Sacramento County. It is worth mentioning that we explored other assumptions without changing substantially the results.

The amount of pesticide available for runoff extraction at the beginning of each storm is defined as “mass available” (*M_avail_pest_*). Starting from the time series obtained from the PUR database, *M_avail_pest_* is calculated as the cumulative applied mass of active ingredient plus the mass available from previous days decreased due to degradation (Equation (4)). If a rainfall event is occurring, *M_avail_pest_* is equal to the mass applied on that specific day, plus the degradated mass available in previous days, minus the amount lost to runoff (Equation (5)):

(4) $$M_{avail\_pest}\left(t_{1}\right)=M_{app\_pest}\left(t_{1}\right)+M_{avail\_pest}\left(t_{0}\right)\cdot e^{-k_{deg}\cdot t}$$

(5) $$M_{avail\_pest}\left(t_{2}\right)=M_{app\_pest}\left(t_{2}\right)+M_{avail\_pest}\left(t_{1}\right)\cdot e^{-k_{deg}\cdot t}-M_{w\_pest}\left(t_{1}\right)$$

where $M_{avail\_pest}\left(t_{1}\right)$ is the mass of pesticide available to be washed off on day one, $M_{avail\_pest}\left(t_{0}\right)\cdot e^{-k_{deg}\cdot t}$ is the degradation of the insecticide mass available to runoff extraction applied the previous day (day zero), *t* is elapsed time in days, $M_{app\_pest}\left(t_{1}\right)$ is the mass applied on day one. If a rainfall event is occurring, $M_{w\_pest}\left(t_{1}\right)$ represents the pesticide mass washed off. The *k_deg_* (t^−1^) value used in this work is the mean of the values calculated by Jorgenson et al. [18] for concrete surfaces.

**Figure 2:**
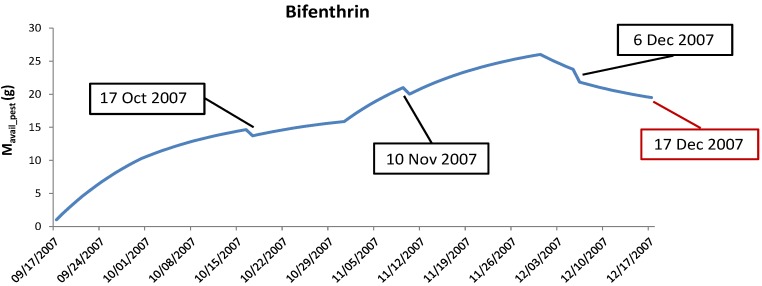
Assessment of bifenthrin *M_avail_* for the winter-event 17 December 2007 (F1) (red label). The three-black labeled dates represent the antecedent three rainfall events.

A daily *M_avail_pest_* was evaluated for each pyrethroid in each basin taking into account the three months immediately preceding each rainfall event. On one side, taking into account the first rain events after the dry season, the choice of considering the antecedent three months follows the studies of Jiang et al. [19] and Richard et al. [20], in which they observed a higher pyrethroid accumulation on exterior residential surfaces during the summer months coupled with warm and dry weather. On the other side, considering the rain events that occur during the wet season (e.g., in December), the choice of considering the previous three months is still applied since the degradation of pyrethroid summer-accumulation due to winter-wash-off is well represented in Equation (5) $(-M_{w\_pest}\left(t\right)$). In Figure 2, we present the application of this concept to bifenthrin for the winter-event that occurred on 17 December 2007 in F1.

It is worth mentioning that the variables *M_w_pest_* and *M_avail_pest_* related to the three pyrethroids are time-dependent (as explained in Equations (4) and (5)). The chosen value of *M_avail_* for each rainfall event is the one calculated at the beginning of the event in absence of rain. In particular, in Figure 2, *M_avail_* for event 17 October 2007 was evaluated with Equation (4) because no rainfall events occurred in the previous three months. Whereas, for event 17 December 2007, Equation (5) was used because in the previous three months three rainfall events occurred.

### 2.3. Data Analysis

Data analysis was carried out using a common multivariate data analysis technique, principal component analysis (PCA). It was run in R (version 3.4.2 R Foundation for Statistical Computing, Vienna, Austria, [21]), by using the libraries “devtools” and “ggbiplot”. This technique was chosen because it offers a holistic vision of all the variables involved in the system. In particular, instead of limiting the analysis to a “macro-description” of the system given by the physical meaning of each original variable, PCA supports a transition to a superior semantic level, a sort of “meta-description” of all the variables involved, given by new variables able to gather possible emerging properties of the system. PCA has been used as a pattern recognition technique in numerous water-quality research studies (e.g., [22]). This analytical approach is capable of clustering similar data together while identifying relationships among variables. In particular, in its graphical representation (PCA biplot), vectors representing parameters that form an acute angle are considered as correlated parameters, while those that are perpendicular are considered as uncorrelated. A detailed description of PCA and its applications can be found in the literature [23].

### 2.4. Model Implementation

The EPA’s Storm Water Management Model (SWMM) numerically obtains the hydrograph and the pollutograph for a storm event (for single and/or sequential events) on the basis of rainfall (hyetograph) and other meteorological inputs (snow, wind) and system characteristics (catchment, conveyance, and storage/treatment) [24,25]. The model comprises functional blocks, which are coordinated by an executive block. These can be sequentially or separately activated, depending on the needs of the user [26]. The runoff and transport blocks were used for this study. Quality simulation processes, in the same unit, include generation of surface runoff constituent loads through the build-up of pollutants during dry weather and wash-off during wet weather. By using inlet hydrographs and pollutographs generated from the runoff unit, the transport block performs the detailed flow and contaminant routing through the sewer system. Flow routing is accomplished using the dynamic wave method, whereas mass conservation is applied for quality processes including first-order decay [27]. The water losses taken into account in this work are represented by the depression storage on the impervious portion of the basins and the infiltration process. The values of depression storage-depth were calculated on the basis of formulations provided byASCE [28]. The infiltration process was modeled by evaluating, for each subcatchment, the percentage of impervious area obtained from the land use map. The infiltration model utilized in this work is based on Horton’s equation, with parameter values chosen according to the typical values reported in the literature, in relation to soil type [29].

## 3. Results and Discussion

### 3.1. Multivariate Data Analysis

An initial analysis was conducted to identify appropriate rainfall and water quality characteristics to prevent correlated parameters from overshadowing critical relationships between rainfall characteristics and the wash-off process. Rainfall characteristics considered in the initial analysis were the antecedent dry period (*ADP*), total rainfall (*Tot_Rain*), and runoff volume (*Runoff_Vol*). Water quality characteristics considered were the pesticide mass available to be washed off (*M_avail_pest_*), and the EML for TSS and each of the pyrethroids under analysis. It is worth noting that both dry and wet periods are captured by the variables chosen; among the rainfall characteristics, *ADP* symbolizes the dry period, while *Tot_Rain* and *Runoff_Vol*, which represent the input and the output of the hydraulic/hydrologic part, respectively, characterize the wet period. Among the water quality characteristics, *M_avail_pest_* can be related to the duration of the dry period, while pollutant EMLs represent the wet period.

**Figure 3:**
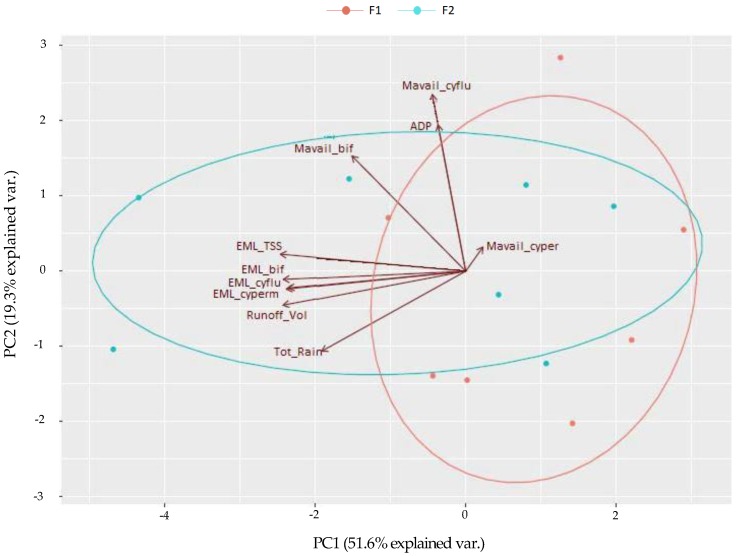
Principal component analysis PCA biplot of F1 and F2 for identifying relationships between rainfall and water quality characteristics.

A data matrix (17 × 11) was the input for the PCA analysis. The objects were the 17 monitored rainfall events while the 11 variables were site location; *ADP*; *Tot_Rain*; *Runoff_Vol*; *M_avail_* of bifenthrin, cyfluthrin, and cypermethrin; and EML of TSS, bifenthrin, cyfluthrin, and cypermethrin. The first two principal components (PCs) were selected since they represented 71% of the variance. In Figure 3, the resulting PCA biplot is shown.

The scores plot summarizes the behavior of the objects in the two components and their similarities. In Figure 3, the scores plot shows two clusters, one for the data points from F1 and the other for the data points from F2. The intersection of these clusters would be expected since, as already explained, F1 and F2 are adjacent urban watersheds characterized by very similar rainfall and catchment characteristics. It is noteworthy that of the 17 data points, 14 are represented in the scores plot since they have no missing data.

The loadings plot analyzed the role of all the variables in the two PCs chosen, their correlations and their importance in the system. Considering the rainfall-related variables, the loadings plot presented in Figure 3 shows reasonable results. A robust relationship between *Tot_Rain* and *Runoff_Vol* was found, while the dry period, represented by *ADP*, is largely independent of the previous two variables. Among the water quality-related variables, all of the *M_avail_* vectors are strongly correlated with *ADP* while the EML vectors are highly correlated with rainfall characteristics. These outcomes confirm that the two processes of pollutant build-up and wash-off from residential surfaces are primarily related to the dry and wet period characteristics, respectively. The most significant result obtained from this analysis is the robust correlation between EML_TSS and all the pyrethroid EMLs. This relationship suggests that sediment transport plays a significant role in the mobilization of insecticides from pervious and impervious surfaces. Impervious surfaces are not generally cleaned prior to pesticide applications, resulting in direct application of active ingredient to particulate matter on the surface. The hydrophobicity of pyrethroid insecticides further promotes particle association. These factors suggest that pre-existing sediments will play a fundamental role in transporting pesticides offsite via surface runoff, as revealed in Figure 3. This finding highlights the need for an in-depth investigation of the relationship between sediments and insecticides, with the aim of developing and testing a new mathematical model able to simulate pyrethroid wash-off based on TSS wash-off at basin-scale.

### 3.2. Relationship between Sediment Particles and Pyrethroids

On the basis of PCA outcomes, an in-depth investigation of the relationship between sediment load washed off and insecticide load washed off was performed. A robust linear correlation was observed between EML_TSS and the EMLs for bifenthrin, cyfluthrin, and cypermethrin (coefficients of determination, R^2^ respectively equal to 0.87, 0.73, and 0.70; see figure in Supplementary Materials (SM-3)).

**Figure 4:**
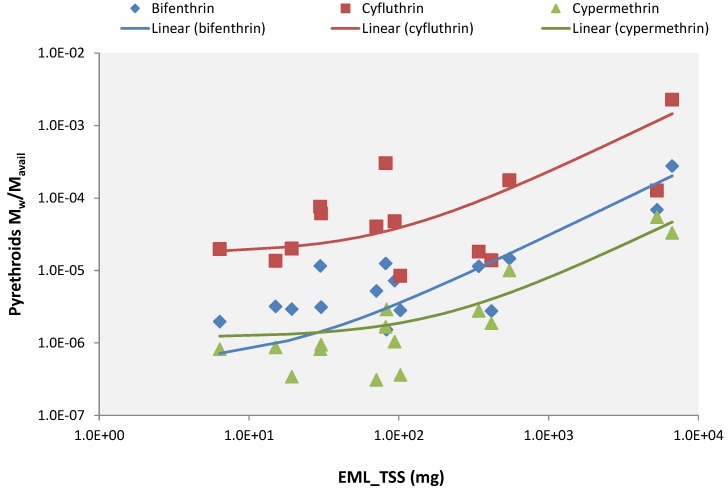
Linear correlation between total suspended solid (TSS) event mean load (EML) and the fraction of insecticide load washed off and mass available to be washed off.

Since some pesticide wash-off models use the fraction of mass washed off divided by the mass available (*M_w_/M_avail_*) instead of the mass itself [18], the relationship between this ratio and the sediment EML was investigated too. In this case, as well, a strong linear correlation between these two parameters was found, showing an R^2^ equal to 0.80 for bifenthrin, 0.60 for cyfluthrin and 0.84 for cypermethrin (Figure 4). These outcomes confirm previous small-scale experiments indicating that 80% of pyrethroids washed off of urban surfaces in rainfall simulation experiments were associated with suspended particles [10].

**Figure 5:**
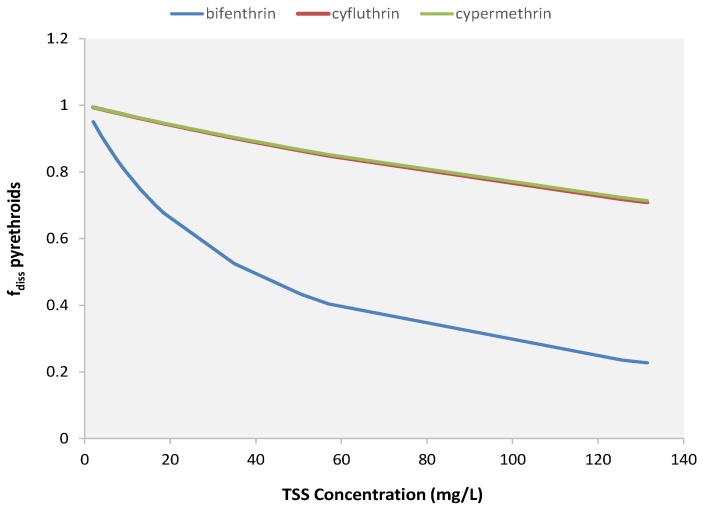
Correlation between TSS concentration and the fraction of dissolved pyrethroids.

Furthermore, the plot in Figure 4 shows an interesting behavior of the three regression curves: by considering EML_TSS = 10 mg as a threshold, the plot can be divided into two parts: left and right portions. In the left part, where the sediment mass is small, the three curves are approximately constant (quasi-parallel to the horizontal axis), demonstrating that all the insecticide mass available to runoff extraction is, then, washed off by the runoff. This behavior suggests that, in this part of the graph, the dissolution process plays a significant role in the transport of pesticides from residential surfaces. In the right portion of the plot, the three regression curves are not constant anymore, but rather begin to increase: the higher the amount of sediment washed off, the bigger the increment of the curves. This behavior reveals that, in the presence of a sufficient amount of particulate matter on the surface, transport of adsorbed compounds begins to eclipse transport of the dissolved compounds. To investigate this assumption, the fraction of pyrethroids dissolved (*f_diss_*) was calculated (see Supplementary Materials (SM-4) for more information about the calculation of *f_diss_*) and plotted against sediment concentration. From the graph presented in Figure 5, it is possible to note that when the TSS concentration is low, a large fraction of insecticide is dissolved, and this value decreases with the increase of the sediment concentration. Even though the bifenthrin *f_diss_* values are lower than those calculated for cyfluthrin and cypermethrin, they are in agreement with the partitioning coefficient (*K_d_*) values estimated by Jiang and Gan [10] for professional concentrate.

These findings emphasize the robust relationship between sediments and insecticides, providing fresh evidence that pre-existing particles on urban surfaces are the primary carriers of hydrophobic pesticide residues in offsite transport from impervious surfaces. This is consistent with a previous study, which observed that, although the concrete slabs were washed with a hose prior to pesticide application, the majority of synthetic pyrethroids in the runoff was consistently found on particles regardless of formulation type [10]. Our findings also confirm previous works showing the ubiquitous presence of relatively high concentrations of pyrethroid insecticides on outdoor urban dust [19] and the high fraction of washed off pesticide that is associated with impervious surfaces where the fine particles primarily accumulate [8]. This proves the need for a new physically based model able to predict pyrethroid wash-off based on TSS wash-off.

### 3.3. Conceptualization of a New Stormwater Quality Model

**Figure 6:**
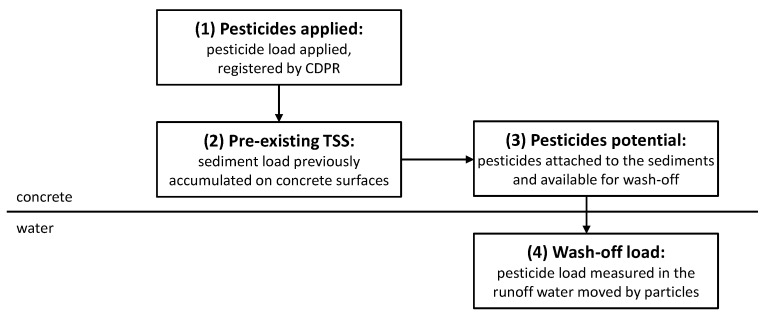
Schematic representation of the new pesticide build-up and wash-off model from residential surfaces.

A new mathematical modeling approach was developed in this study to characterize and predict pyrethroid wash-off profiles from urban surfaces. In Figure 6, we show a schematic representation of the new model. Once applied to residential surfaces (1), a certain amount of pyrethroid goes directly to the surfaces, whereas the remaining part deposits on pre-existing sediment particles (2). The portion of pesticides adsorbed to particles represents the pesticide potential (3); e.g., the part of pyrethroids available for runoff extraction. If a rainfall event occurred, a certain amount of pesticide load that was attached to sediments could be measured in the runoff water (4).

**Figure 7:**
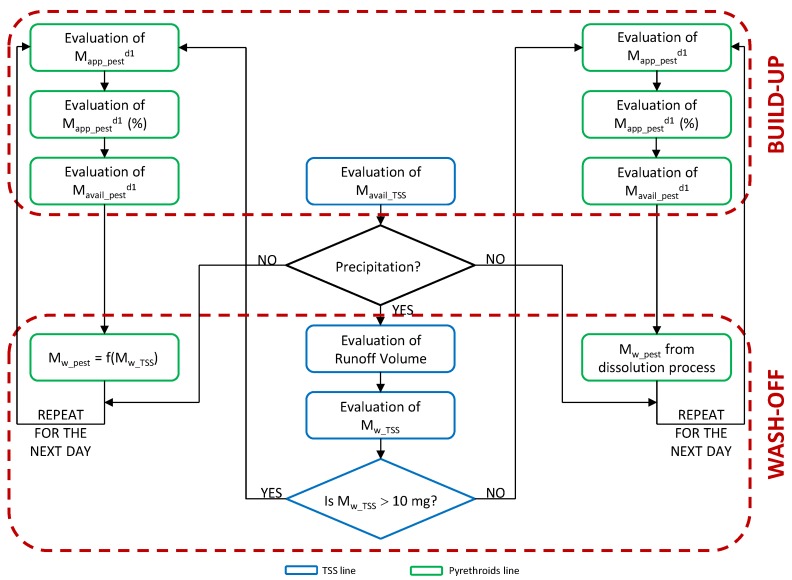
Flow chart of the new physically based stormwater quality model (d1 = “day one”).

The conceptualization of this new modeling approach was developed by taking into account not only the robust correlation that exists between pyrethroid mass and sediment load, but also the threshold between dissolution and adsorption process control represented by the TSS mass washed off (*M_w_TSS_*) equal to 10 mg. The flow chart of the new physically-based model is shown in Figure 7. It is noteworthy that this new modeling approach, even though it was designed with a simplified fixed threshold (10 mg), represents a first effort that overcomes the significant limitation that characterizes the previous methodologies developed for the estimation of pesticide runoff potential; i.e., they do not separate dissolved and particle-bound offsite pyrethroid transport [10]. Based on this approach, future research is needed to develop a more accurate “range of TSS mass-values” to substitute to the 10 mg threshold. A range of values, instead of a fixed value, would better represent the transition from dissolution to adsorption process.

The model is characterized by two main lines: the TSS line, represented by blue blocks, and the pyrethroid line, symbolized by green blocks. The two red dotted blocks include all the steps for simulating the two primary pollutant processes commonly replicated in these mathematical models: build-up and wash-off. The development of a hydraulic/hydrologic model capable of predicting the quantity response of F1 and F2 to a diverse set of inputs (*Evaluation of Runoff Volume*), and the simulation of sediment build-up (*Evaluation of M_avail_TSS_*) and wash-off (*Evaluation of M_w_TSS_*), were issues successfully tackled in our previous work [30] (see Supplementary Materials (SM-5) for more information about the hydrologic and sediment simulations). By exploiting these outcomes, for the current model, it is necessary to check if the sediment load washed off is higher than 10 mg. If not, the pesticide wash-off process has to be simulated by considering the dissolution process as the primary mechanism by which pyrethroid insecticides are transported away from their point of application (*M_w_pest_* from dissolution process). This process is not involved in the simulation domain of this new modeling approach since it was already developed by previous researchers [18,31]. If instead, the sediment load washed off is higher than 10 mg, pesticide wash-off is simulated as a function of TSS wash-off (*M_w_pest_ = f*(*M_w_TSS_*)). By developing this relationship, in this study we are analyzing and testing at basin-scale, the idea that particles are the principal vector carrying pyrethroid residues from pervious and impervious surfaces into runoff water at higher TSS loads. The surface-wiping method tested by Jiang and Gan [10] is a simple, non-invasive method for estimating the runoff-transferable pesticide residues from impervious residential surfaces. However, this method does not differentiate between pesticide wash-off in dissolved and particle-bound forms. The novelty of our modeling approach is represented not only from the larger scale and database used, but also for the fact that, to our knowledge, this model is the first attempt to separately predict the dissolved and particle-bound offsite transport potential of pesticides from residential surfaces via runoff. 

### 3.4. Insecticide Simulations

**Figure 8:**
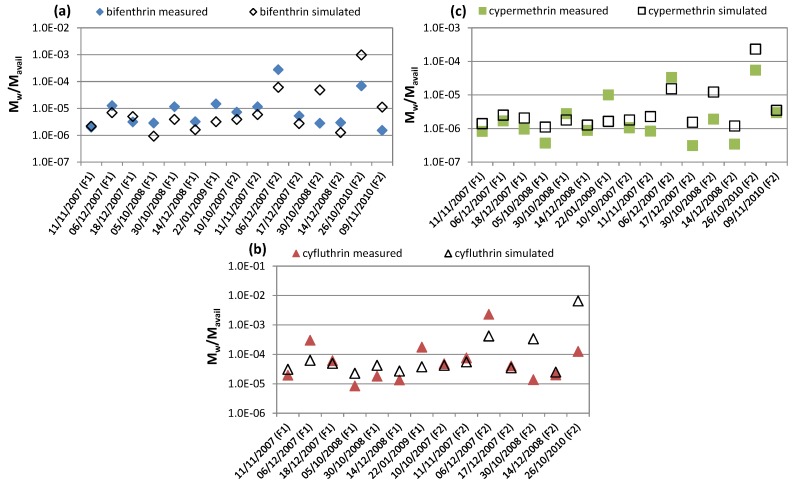
Comparison between the measured and simulated fraction of mass washed off and mass available to wash-off of (**a**) bifenthrin, (**b**) cyfluthrin, and (**c**) cypermethrin.

With the aim at predicting pyrethroid wash-off on impervious surfaces at watershed-scale, the first set of simulations was launched by exploiting the linear correlation found between TSS EML and the fraction of insecticide load washed off and mass available to be washed off (presented in Figure 4). The comparison between measurements and predictions for bifenthrin, cyfluthrin, and cypermethrin are shown in Figure 8, where it is possible to see the accuracy and reliability of the model used.

**Figure 9:**
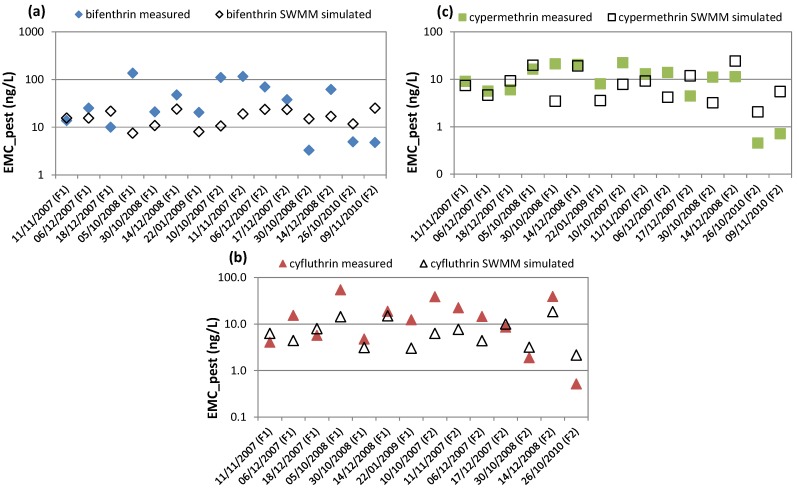
Comparison between observed and predicted Event Mean Concentration (EMC) of (**a**) bifenthrin, (**b**) cyfluthrin, and (**c**) cypermethrin. The predictions are obtained with Storm Water Management Model (SWMM).

The second set of simulations was developed, by using the robust linear correlation found between EML TSS and EML of the three pyrethroids (reported in Supplementary Materials (SM-3)). The results obtained from the simulations were graphically compared with the observations, for all the rainfall events monitored at F1 and F2. Since the comparisons were very similar to the ones shown in Figure 8, these plots are presented in Supplementary Materials (SM-6). Afterward, this last linear correlation that connects EMLs was transformed into a relationship between EMCs, by using Equation (2) and, then, was implemented in SWMM. A graphical and numerical comparison between the last simulations obtained and the measured EMC and EML of bifenthrin, cyfluthrin, and cypermethrin was carried out. For brevity, in Figure 9 we present only the graphical comparison between EMCs, while Table 1 shows EMCs and EMLs statistical comparisons. The latter were made by evaluating the root mean square error (RMSE), Nash–Sutcliff efficiency (NSE), and Kling–Gupta efficiency (KGE) for each rainfall event (Equations (6)–(8)):

(6) $$\mathrm{RMSE}=\sqrt{\frac{\sum_{i=1}^{N}{\left(x_{obs}\left(t\right)-x_{sim}\left(t\right)\right)}^{2}}{N}}$$

(7) $$\mathrm{NSE}=1-\frac{\sum_{t=1}^{N}{\left[x_{obs}\left(t\right)-x_{sim}\left(t\right)\right]}^{2}}{\sum_{t=1}^{N}{\left[x_{obs}\left(t\right)-\mu_{obs}\right]}^{2}}$$

(8) $$\mathrm{KGE}=1-\sqrt{{\left(r-1\right)}^{2}+{\left(\alpha -1\right)}^{2}+{\left(\beta -1\right)}^{2}}$$

where $x_{obs}\left(t\right)$ is the observed value at the time step t, $x_{sim}\left(t\right)$ is the simulated value, $\mu_{obs}$ is the mean of the observed values over the entire simulation period of length *N*, *r* is the linear correlation coefficient between $x_{obs}$ and $x_{sim}$, and being $\left(\mu_{sim},\sigma_{sim}\right)$ and $\left(\mu_{obs},\sigma_{obs}\right)$ the first two statistical moments (means and standard deviations) of $x_{sim}$ and $x_{obs}$ respectively, the quantity α is a measure of relative variability in the simulated and observed values ($\alpha =\sigma_{sim}/\sigma_{obs}$), β is the bias ($\beta =\mu_{sim}/\mu_{obs}$).

From the graphical and numerical outcomes, it is possible to understand that this new modeling approach is capable of providing accurate and reliable predictions of the offsite transport of pyrethroid residues from residential surfaces at basin-scale. In particular, considering how wide the goodness-of-fit-range of variations are (RMSE∈[0,+∞), NSE∈(−∞,1], and KGE∈(−∞,1]), we believe that the values obtained in this study can be considered satisfactory. The strong foundation on which this model is built is represented by the numerous experimental results proving that particles play a critical role in the offsite transport of runoff-borne pyrethroid residues from residential surfaces [8,10,19,20]. The new modeling approach also deals in a great way with the challenges of large database and watershed-scale modeling resulting from the uncertainty in the input parameters (e.g., mass of pesticides applied on the surface, cleaning of the surfaces, amount of pesticide available to runoff extraction, etc.). Moreover, this model tackles, for the very first time, the prediction of urban pesticide runoff from residential surfaces by separately considering dissolved and particle-bound forms. It also incorporates a typical land-cover of a low and very-low-density residential area; therefore, it is highly applicable to other residential areas in California, which includes a high percentage of urbanized area.

## 4. Conclusions

To better understand the wash-off process of pesticide residues in urban runoff, the association of pyrethroid residues with sediments in runoff water from residential surfaces in two watersheds located in Northern California (Sacramento County) was investigated. Multi-year monitoring data provided records of stormwater quality (bifenthrin, cyfluthrin, cypermethrin and sediment EMC), stormwater quantity (flow rate) and rainfall. These data, combined with data on pesticide applications from California’s pesticide use database, were analyzed with PCA to define relationships among pesticide accumulation and runoff, different rainfall characteristics, and sediment build-up and wash-off processes. The most significant result from the PCA is the identification of a robust correlation between TSS mass washed off and pyrethroid mass washed off, shown from numerous experimental results.

Based on PCA outcomes, an in-depth investigation of the relationship between TSS and insecticide load washed off was carried out. In particular, a strong linear correlation between sediments and each pesticide load was found (R^2^ never lower than 0.6). Furthermore, it was found that the value of TSS load equal to 10 mg represents a threshold between the two primary mechanisms that cause the removal of insecticides from their point of application: pesticide dissolution and the mobilization of pesticides adsorbed to solid particles.

On the basis of these outcomes, a new mathematical modeling approach was developed here to predict, at basin-scale, pesticide wash-off from residential surfaces as a function of TSS wash-off. The simulation range includes the case in which sediment load washed off is greater than 10 mg. The case in which TSS mass is lower than 10 mg is handled using approaches developed by previous researchers. The accuracy and precision of this new water quality model were estimated by evaluating, for all the pyrethroid EMLs, RMSE, NSE, and KGE that are respectively equal to 0.13, 0.28, and 0.56 on average. Based on this approach, future research is needed to develop a more accurate “range of TSS mass-values” to substitute to the 10 mg threshold. A range of values, instead of a fixed value, would better represent the transition from dissolution to adsorption process.

By assessing model performance and interpreting model parameters, this study provides the first comprehensive approach for the numerical prediction of pesticide wash-off from residential surfaces explicitly accounting for sediment wash-off; further, it is the first to be applied at field scale. Modeling scenarios with a typical rainfall pattern and concrete surface condition could be developed as guidelines for the wash-off simulations and model application. On the basis of appropriate modeling scenarios and calibrated parameters, the model developed in this study is anticipated to provide reasonable estimates of pesticide wash-off to contribute to the quantification of pesticide loads in urban environmental settings, and inform appropriate mitigation policies to control those risks.

## Figures and Tables

**Table 1:** Numerical comparison between the simulated and measured EMCs and EMLs for each rainfall event.

	RMSE	NSE	KGE
**EMC**	RMSE_bif_ = 52.945	NSE_bif_ = −0.535	KGE_bif_ = −0.61296
	RMSE_cyfl_ = 16.154	NSE_cyfl_ = −0.060	KGE_cyfl_ = 0.041207
	RMSE_cyperm_ = 8.106	NSE_cyperm_ = −0.433	KGE_cyperm_ = 0.28582
	RMSE_tot_ = 32.574	NSE_tot_ = −0.481	KGE_tot_ = −0.19214
**EML**	RMSE_bif_ = 0.213	NSE_bif_ = 0.296	KGE_bif_ = 0.558342
	RMSE_cyfl_ = 0.050	NSE_cyfl_ = 0.073	KGE_cyfl_ = 0.35206
	RMSE_cyperm_ = 0.047	NSE_cyperm_ = 0.054	KGE_cyperm_ = 0.346344
	RMSE_tot_ = 0.131	NSE_tot_ = 0.279	KGE_tot_ = 0.556032

RMSE: Equation (6); NSE: Equation (7); KGE: Equation (8).

## Supplementary Materials

The following are available online at http://www.mdpi.com/1660-4601/15/7/1464/s1. Content: SM-1: Summary of the rainfall/runoff data for all events for F1 and F2. SM-2: Summary of TSS and pyrethroid EML values for all events for F1 and F2. SM-3: Linear correlation between TSS load washed off and insecticide load washed off. SM-4: Calculation of the fraction of pyrethroids dissolved (*f_diss_*). SM-5: Hydraulic/hydrologic model and sediment simulation. SM-6: Comparison between measured and simulated mass washed off of (a) bifenthrin, (b) cyfluthrin, and (c) cypermethrin.
