# Supplementary material for: Role of Sediments in Insecticide Runoff from Urban Surfaces: Analysis and Modeling
Source: Int J Environ Res Public Health. 2018 Jul 11;15(7):1464. doi: 10.3390/ijerph15071464 (PMC6069241; doi:10.3390/ijerph15071464)

Supplementary Materials

Role of Sediments in Insecticide Runoff from Urban Surfaces: Analysis and Modeling

Angela Gorgoglione [1], Fabián A. Bombardelli [1], Bruno J.L. Pitton [2], Lorence R. Oki [2], Darren L. Haver [3], and Thomas M. Young [1,*]

[1] Department of Civil and Environmental Engineering, University of California, Davis, One Shields Avenue, Davis, 95616 California, USA; agorgoglione@ucdavis.edu, fabombardelli@ucdavis.edu, tyoung@ucdavis.edu

[2] Department of Plant Sciences, University of California, Davis, One Shields Avenue, Davis, 95616 California, USA; bjpitton@ucdavis.edu, lroki@ucdavis.edu

[3] Division of Agriculture and Natural Resources, South Coast Research & Extension Center, University of California, Irvine, 7601 Irvine Blvd., Irvine, 92618 California, USA; dlhaver@ucanr.edu

* Correspondence: tyoung@ucdavis.edu; Tel.: +1 (530) 754-9399

Content:

SM-1: Summary of the rainfall/runoff data for all events for F1 and F2.

SM-2: Summary of TSS and pyrethroid EML values for all events for F1 and F2.

SM-3: Linear correlation between TSS load washed off and insecticide load washed off.

SM-4: Calculation of the fraction of pyrethroids dissolved (f_{diss}).

SM-5: Hydraulic/hydrologic model and sediment simulation.

SM-6: Comparison between measured and simulated mass washed off of (a) bifenthrin, (b) cyfluthrin, and (c) cypermethrin.

SM-1: Summary of the rainfall/runoff data for all events for F1 and F2.

Basin	Events	Total Rainfall (mm)	Event Duration (hr)	Antecedent Dry Period (day)	Runoff Volume (m^3)	Runoff Peak (m^3/s)	Total Depth (m)
**F1**	10/17/2007	1.5	0.90	-	1023.44	0.08	86.29
	11/11/2007	41.3	17.13	24	3191.99	0.30	131.01
	12/06/2007	79.7	38.83	24	11693.45	1.18	224.74
	12/16/2007	49.2	46.07	9	6070.39	0.18	203.73
	10/05/2008	13.3	13.63	-	817.74	0.24	70.49
	10/30/2008	49.6	106.10	23	9780.47	0.52	416.15
	12/14/2008	6	21.47	39	1157.13	0.11	85.81
	01/22/2009	35.9	97.67	35	9626.53	0.32	387.53
	09/22/2014	13.2	6.27	-	971.70	0.25	170.20
	10/25/2014	2.7	2.52	30	393.81	0.11	96.86
	10/31/2014	16.2	28.27	5	1904.97	0.26	225.92
**F2**	10/10/2007	18.20	4.87	-	1802.94	0.31	63.03
	10/17/2007	1.50	0.87	5	9477.00	0.04	39.91
	11/11/2007	41.30	17.10	24	3149.45	0.23	116.38
	12/06/2007	79.40	30.43	24	132520.38	5.40	532.29
	12/17/2007	49.50	56.10	9	3831.24	0.13	170.73
	10/30/2008	84.90	98.07	23	30237.43	1.58	484.17
	12/14/2008	18.60	22.57	39	1170.94	0.14	98.22
	12/16/2008	6.30	12.17	1	498.84	0.11	29.68
	21/12/2008	28.90	20.87	5	2710.86	0.21	109.49
	05/10/2010	11.60	2.07	-	1005.66	0.59	19.84
	05/25/2010	9.30	4.30	14	830.79	0.17	23.56
	10/26/2010	106.60	32.80	-	662453.15	7.52	2382.54
	11/09/2010	24.80	11.33	10	20715.56	0.64	250.33
	10/04/2011	14.80	10.00	-	2366.42	0.28	10.64
	10/10/2011	18.80	15.20	4	3740.74	0.32	15.03
	10/21/2012	28.80	12.50	-	2826.23	0.27	11.09
	09/25/2014	15.30	5.50	-	1058.15	0.21	4.32
	10/25/2014	0.90	0.58	29	38.21	0.08	5.74
	10/31/2014	21.80	13.50	5	1931.07	0.21	9.60
	11/12/2014	6.70	11.50	10	379.57	0.03	6.13

SM-2: Summary of TSS and pyrethroid EML values for all events for F1 and F2.

Basin	Events	TSS EML (mg)	Bifenthrin EML (ng)	Cyfluthrin EML (ng)	Cypermethrin EML (ng)
**F1**	17/10/2007	**NA**	9.19E+06	**NA**	**NA**
	11/11/2007	6.384	4.41E+07	1.31E+07	2.89E+07
	12/06/2007	81.854	2.95E+08	1.80E+08	6.56E+07
	12/18/2007	30.352	6.07E+07	3.49E+07	3.65E+07
	10/05/2008	102.626	1.12E+08	4.46E+07	1.34E+07
	10/30/2008	342.317	2.06E+08	4.66E+07	2.06E+08
	12/14/2008	15.043	5.53E+07	2.18E+07	2.37E+07
	01/22/2009	548.712	1.97E+08	1.20E+08	7.75E+07
**F2**	10/10/2007	93.753	1.99E+08	6.97E+07	4.02E+07
	17/10/2007	**NA**	2.24E+08	6.12E+07	4.93E+07
	11/11/2007	29.92	3.66E+08	7.07E+07	4.10E+07
	12/06/2007	6692.279	9.25E+09	1.93E+09	1.85E+09
	12/17/2007	70.878	1.45E+08	3.29E+07	1.69E+07
	10/30/2008	415.765	1.00E+08	5.75E+07	3.35E+08
	12/14/2008	19.321	7.26E+07	4.59E+07	1.33E+07
	10/26/2010	5299.625	3.29E+09	3.44E+08	2.98E+08
	11/09/2010	82.862	9.90E+07	**NA**	1.47E+07

SM-3: Linear correlation between TSS load washed off and insecticide load washed off.

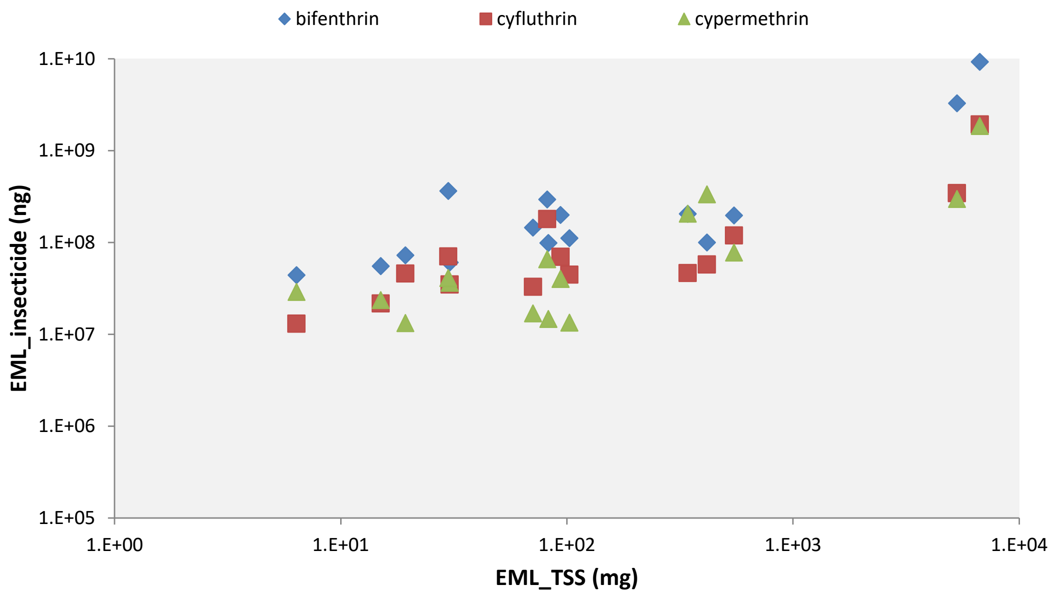

Bifenthrin: $y = 1.0E^{+06}x - 4.0E^{+07}$ with $R^2 = 0.87$

Cyfluthrin: $y = 1.9E^{+05}x + 2.0E^{+07}$ with $R^2 = 0.73$

Cypermethrin: $y = 1.8E^{+05}x + 3.1E^{+07}$ with $R^2 = 0.70$

where x and y represent EML_TSS (mg) and EML of each insecticide (ng) respectively.

SM-4: Calculation of the fraction of pyrethroids dissolved (f_{diss}).

By considering TSS concentration (C_{TSS}), the concentration of pesticide dissolved (C_{diss_pest}), and the concentration of pesticide adsorbed to solid particles (C_{ads_pest}), the distribution coefficient between water and sediments of pesticides (K_d) is given by:

$$K_d = \frac{C_{ads\_pest}}{C_{diss\_pest}} \left[\frac{\mu g_{pest} / g_{TSS}}{\mu g_{pest} / L_w} \right] \qquad \text{(I)}$$

Considering (I), it is possible to calculate the fraction of pyrethroids dissolved (f_{diss}):

$$f_{diss} = \frac{C_{diss\_pest} \cdot V_w}{C_{diss\_pest} V_w + C_{ads\_pest} C_{TSS}} = \frac{C_{diss\_pest}}{C_{diss\_pest} + K_d C_{diss\_pest} \cdot C_{TSS}} = \frac{1}{1 + K_d \cdot C_{TSS}} \qquad \text{(II)}$$

knowing that: $K_d = f_{OC} \cdot K_{OC}$

By assuming the sediment organic carbon content (f_{OC}) equal to f_{OC}=0.05 for bifenthrin, cyfluthrin, and cypermethrin, and knowing that the organic carbon–normalized coefficient (K_{OC}) of the three pyrethroids is $K_{OC\_bif}=1.31 \cdot 10^5 \div 3.02 \cdot 10^5$, $K_{OC\_cyfl}=6.24 \cdot 10^4$; $K_{OC\_cyperm}=6.10 \cdot 10^4$, it was possible to evaluate K_d.

SM-5: Hydrologic model and sediment simulation.

The hydrologic and water-quality (sediment) models were implemented in the Storm Water Management Model (SWMM). Appropriate model parameter values were identified to generate separate models capable of predicting the hydrologic and water-quality responses of F1 and F2 to a diverse set of inputs.

Hydrologic simulations

To simulate the runoff from urban surfaces, the dynamic-wave equation was chosen. Furthermore, the water losses taken into account are represented by the depression storage on the impervious portion of the basin and the infiltration process. The latter was modeled by evaluating, for each subcatchment, the percentage of impervious and pervious area obtained from the land-use map. The infiltration model utilized in this work was based on Horton's equation, whose parameter values have been chosen according to the representative values reported in the literature, in relation to soil type.

Eight parameters of the runoff block of SWMM were used to calibrate the hydraulic-hydrologic model: the depth of depression storage on impervious (*Dstore-Imperv*) and pervious (*Dstore-Perv*) portions of the subcatchment, Manning's coefficient for overland flow over the impervious (*N-Imperv*) and pervious (*N-Perv*) portions of the subcatchment, the percent of impervious area without depression storage (*%Zero Imperv*), and the infiltration parameters of Horton's equation. Working within the established range, and comparing numerically and statistically the simulation with the measured hydrograph, the calibration was performed until a good fit was obtained. For brevity, in Table I only the most recent rainfall events are shown, and, among these, only the event 10/31/2014 related to F1 is reported in Fig. I.

Table I. Numerical comparison between the simulated and measured hydrographs for each rainfall event.

Basin	Events	R^2	RMSE	NSE
**F1**	09/25/2014	0.861	17.941	0.721
	10/31/2014	0.872	13.274	0.747
**F2**	09/25/2014	0.942	52.392	-0.123
	10/31/2014	0.850	43.428	-0.812
	11/13/2014	0.933	12.806	-16.552

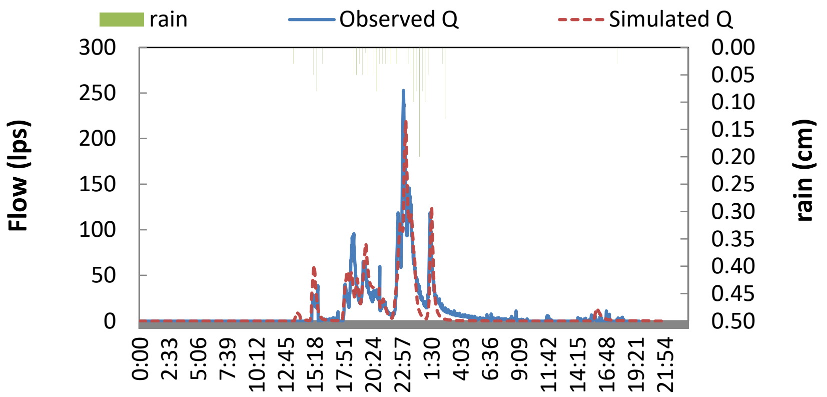

Fig. I. Comparison between observed (blue line) and simulated hydrograph (red dotted line) for the event 31 Oct 2014 at F1.

<u>Sediment simulations</u>

TSS build-up within a land-use category is described by a mass per unit of subcatchment area. The amount of build-up is a function of the number of dry weather days antecedent to the rainfall event. The build-up function follows a growth law that asymptotically approaches a maximum limit:

$$M_a(d_{adp}) = \left(\frac{Accu}{Disp}\right) A\, P_{imp} \left(1 - e^{-(Disp\; d_{adp})}\right) \qquad \text{(III)}$$

where $M_a(d_{adp})$ represents the pollutant build-up during the antecedent dry period [kg/ha]; $Disp$ is the parameter that measures the disappearance of accumulated solids due to the action of wind or vehicular traffic [1/d]; P_{imp} is the impervious area fraction; $Accu$ is the parameter that characterizes the solids build-up rate [kg/(ha d)]; $\left(\frac{Accu}{Disp}\right) A\, P_{imp}$ represents the maximum asymptotic limit of the build-up curve.

The pollutant wash-off over different land uses takes place during wet periods, and it is described by the differential equation:

$$\frac{dM_d(t)}{dt} = -Arra\; i(t)^{wash}\; M_a(t) \qquad \text{(IV)}$$

where $\frac{dM_d(t)}{dt}$ is the wash-off load rate [kg/h]; $Arra$ is the wash-off coefficient [mm^{-1}]; $i(t)$ is the runoff rate [mm/h]; $wash$ is the wash-off exponent, a parameter that controls the influence of rainfall intensity on the amount of leached pollutants.

SWMM calculates the spatial and temporal trend of pollutant concentrations in the drainage network, assuming that the conduits behave as ideal, completely-mixed flow reactors (CMFRs). The control volume (the reactor volume) coincides with the conduit volume. Inside the reactor, the mathematical balance is obtained from a macroscopic material mass balance:

$$\frac{d(VC)}{dt} = Q_{in}C_{in} - Q_{out}C_{out} - kVC_{out} \qquad \text{(V)}$$

in which V represents the water volume in the conduit (reactor), calculated at each time step [m^3]; Qin is the inflow in the conduit [m^3/s]; Cin is the sediment concentration at the inlet of the conduit [mg/L]; Qout is the outflow to the conduit [m^3/s]; Cout is the sediment concentration in volume V at the outlet of the conduit [mg/L]; and k is the decay coefficient [s^{-1}].

Four parameters of the runoff block were identified for the calibration of the water-quality model. For the build-up function: the parameter that characterizes the solids build-up rate (*Accu*) and the parameter that identifies the disappearance of accumulated sediments due to the action of the wind or vehicular traffic (*Disp*). For the wash-off function: the wash-off coefficient (*Arra*) and the wash-off exponent (*wash*). As we did for the hydraulic-hydrologic model, calibration was performed via an iterative process by adjusting the water-quality parameters. The results of the calibration process are shown in Fig. II, which compares the correlation between sediment load observations and simulations.

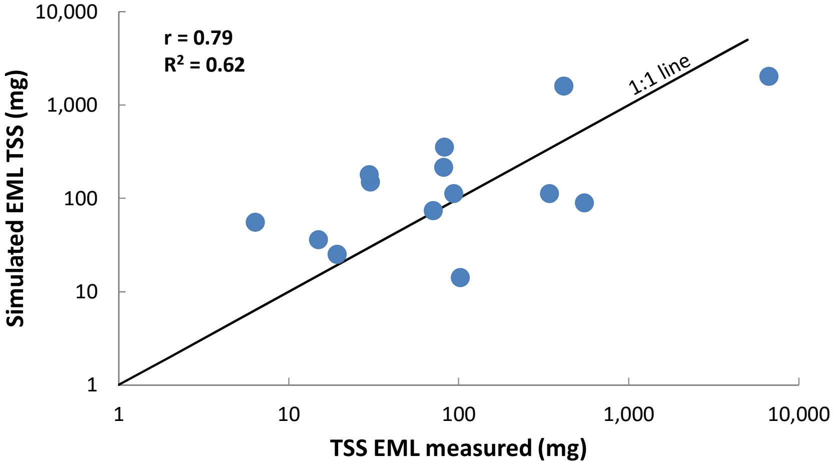

Fig. II. Linear correlation among observed and simulated EML for F1 and F2.

SM-6: Comparison between measured and simulated mass washed off of (a) bifenthrin, (b) cyfluthrin, and (c) cypermethrin.

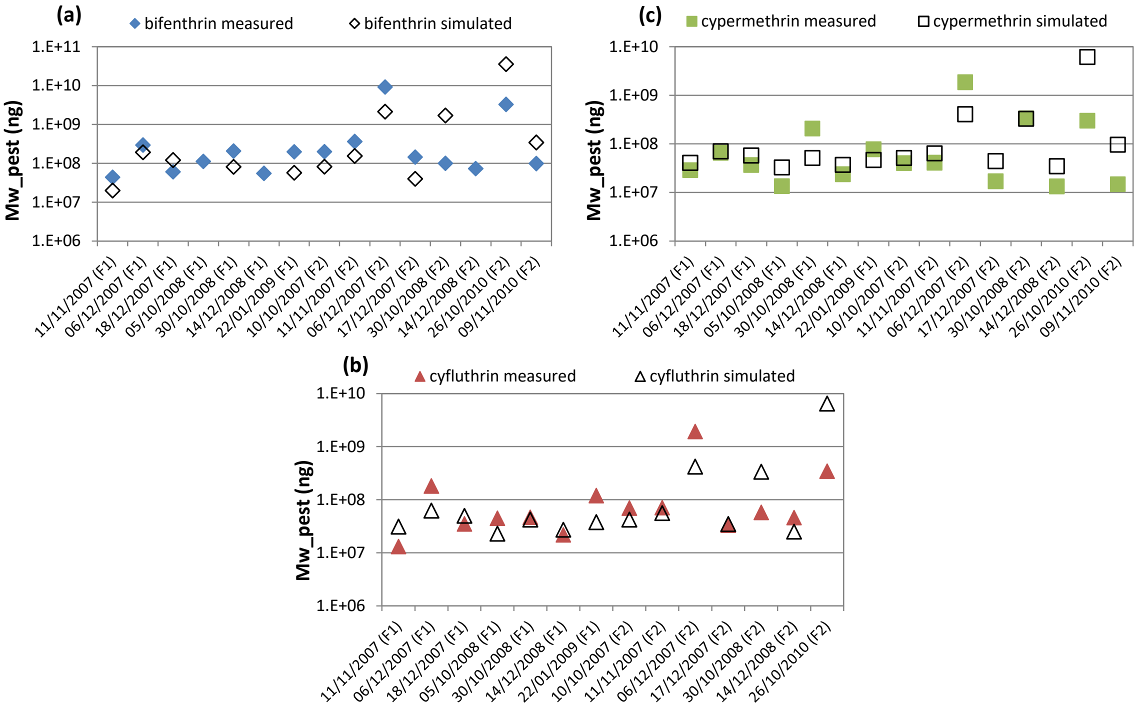

Supplement: Supplementary file 1 [file ijerph-15-01464-s001.pdf]
